# *FRZB* as a key molecule in abdominal aortic aneurysm progression affecting vascular integrity

**DOI:** 10.1042/BSR20203204

**Published:** 2021-01-06

**Authors:** Chang-Kyu Oh, Yeji Ko, Jeong Jun Park, Hye Jin Heo, Junho Kang, Eun Jung Kwon, Ji Wan Kang, Yoonsung Lee, Kyungjae Myung, Jin Mo Kang, Dai Sik Ko, Yun Hak Kim

**Affiliations:** 1 Center for Genomic Integrity, Institute for Basic Science (IBS), Ulsan 44919, Republic of Korea; 2 Department of Statistics, University of Michigan, Ann Arbor, MI, U.S.A.; 3 Department of Anesthesiology and Pain Medicine, Asan Medical Center, University of Ulsan College of Medicine, Seoul, Republic of Korea; 4 Department of Anatomy, School of Medicine, Pusan National University, Yangsan, Republic of Korea; 5 Department of Biomedical Informatics, School of Medicine, Pusan National University, Yangsan, Republic of Korea; 6 Division of Vascular Surgery, Department of Surgery, Gachon University Gil Medical Center, Incheon, Republic of Korea

**Keywords:** abdominal aortic aneurysm, bioinformatics, FRZB, gene expression omnibus, vascular integrity, zebrafish

## Abstract

Abdominal aortic aneurysm (AAA), when ruptured, results in high mortality. The identification of molecular pathways involved in AAA progression is required to improve AAA prognosis. The aim of the present study was to assess the key genes for the progression of AAA and their functional role. Genomic and clinical data of three independent cohorts were downloaded from the National Center for Biotechnology Information (NCBI) Gene Expression Omnibus (GEO) (GSE57691, GSE7084, and GSE98278). To develop AAA diagnosis and progression-related differentially expressed genes (DEGs), we used a significance analysis of microarray (SAM). Spearman correlation test and gene set analysis were performed to identify potential enriched pathways for DEGs. Only the Frizzled-related protein (*FRZB*) gene and chromosome 1 open reading frame 24 (*C1orf24*) exhibited significant down-regulation in all analyses. With *FRZB*, the pathways were associated with RHO GTPase and elastin fiber formation. With *C1orf24*, the pathways were elastic fiber formation, extracellular matrix organization, and cell–cell communication. Since only *FRZB* was evolutionally conserved in the vertebrates, function of *FRZB* was validated using zebrafish embryos. Knockdown of *frzb* remarkably reduced vascular integrity in zebrafish embryos. We believe that *FRZB* is a key gene involved in AAA initiation and progression affecting vascular integrity.

## Introduction

Abdominal aortic aneurysm (AAA) is a late age-of-onset disorder that affects approximately 5% of men aged 65–74 years; when ruptured, AAAs are associated with a mortality rate of up to 90% [1]. Ruptured aortic aneurysms are a major cause of death, and they were reported to be the 13th leading cause of mortality in 2017 [2]. Preemptive elective open surgical or endovascular repair is considered when the AAA diameter is ≥55 mm, when it is rapidly expanding (≥10 mm/year), or when it causes symptoms [3]. There are, however, large intrapatient and interpatient variations with regard to rates of expansion of small AAAs during follow-up [4]. Therefore, increasing demand to improve risk stratification in patients with AAA has been claimed [5,6].

The pathophysiology of AAA has been extensively studied. AAA was previously believed to be a form of atherosclerosis; however, it is now recognized as a distinct degenerative process involving all layers of the vessel wall and is characterized by destruction of elastin and collagen in the media and adventitia and loss of smooth muscle cells with thinning of the media [1,7,8]. However, a lack of knowledge remains regarding the molecular mechanisms that initiate aneurysm formation and expansion. In current clinical practice, the strategy to stratify patients at high risk for aneurysm progression is lacking.

Serial monitoring of biological activity of AAA would be necessary to lower mortality and morbidity from AAA rupture; however, no biomarkers have yet proven to be of prognostic value additive to AAA diameter to predict AAA expansion [9,10]. AAA growth rate can only be acknowledged retrospectively. To be able to find preventive strategies, the molecular mechanisms behind the disease and drug targets need to be evaluated in detail.

Some studies have attempted to evaluate the candidate genes and pathways potentially associated with AAA, but a large portion of their data was prone to bias and focused on pre-selected genes encoding matrix metalloproteinases, components of the immune system, and known atherosclerosis-related factors [8,11–14]. Therefore, to understand the molecular pathways controlling the progression of AAA without bias, we examined the statistical analysis of three cohorts acquired from the National Center for Biotechnology Information (NCBI) Gene Expression Omnibus (GEO) Series (GSE57691, GSE7084, and GSE98278).

## Methods

### Patient data

Microarray gene expression data and clinical information of three different AAA patient cohorts were obtained from three independent sources: James Cook University, Wayne State University School of Medicine, and Ludwig Maximilians University Munich. They were accessed through the NCBI GEO Series (GSE57691, GSE7084, and GSE98278, respectively). GSE57691 and GSE7084 contain both normal and AAA specimen data. Thus, they were evaluated to find the differentially expressed genes (DEGs) among the AAA patient group. On the contrary, GSE98278, which does not include the normal patient data, was used to determine genes that are linked to the further development of AAA. More details of the patient data are shown below (Table 1). Since the data we used were publicly accessible, IRB approval was not required.

**Table 1:** Patients’ characteristics

	GSE57691		GSE7084		GSE98278			
	Non-AAA	AAA	Non-AAA	AAA	Size		Status	
					Intermediate	Large	Stable	Rupture
***n***	10	49	11	10	15	16	31	17
**Age (years)**	68.4 (±4.5)	69.8 (±7.0)*	64.5 (±10.7)	66.4 (±5.7)	67.7(±7.4)	72.3(±9.2)	69.5 (±7.2)	73.5 (±11.3)
**Women (%)**	40.0%	4.1%	27.3%	20.0%	6.7%	12.5%	3.2%	23.5%
**Diameter (mm)**	-	62.3 (±10.2)*	-	-	54.1 (±1.8)	84.1 (±12.6)	62.3 (±12.1)	77.0 (±14.7)
**Hypertension (%)**	-	81.6%	-	-	86.7%	93.8%	93.5%	88.2%
**Diabetes (%)**	-	22.4%	-	-	26.7%	31.3%	32.3%	23.5%
**Dyslipidemia (%)**	-	71.4%	-	-	86.7%	81.3%	77.4%	82.3%
**Coronary heart disease (%)**	-	51.0%	-	-	46.7%	43.8%	58.1%	41.2%
**Ever smoker (%)**	50.0%	57.1%	-	-	33.3%	68.8%	58.1%	58.8%

Nominal variables are presented as percentages; continuous variables are presented as mean ± standard deviation. Asterisk (*) means Cohen’s formula for pooled standard deviation.

### Standardization

In microarray data, each gene has a different range of expression values. For instance, while some gene expression levels range from 0 to 100, others can range from 500 to 1000. Thus, to facilitate comparison between them, we had to standardize their values and to bring all the genes to the same range. Since microarray data from GSE57691 had been distributed after standardization, only gene expression measurements from GSE7084 and GSE98278 needed to be standardized. The standardization process has assigned a standard score (z-score) to each gene expression value in the data. The ‘scale’ function in the ‘base’ package R (version 3.6.1) was used in this step.

### Missing values

There were no missing values from GSE57691, GSE7084, and GSE98278. However, a huge portion of genes within the three datasets appeared repetitively in different columns. In order to organize these recurring genes, we aggregated the genes in the columns by mean, using the ‘aggregate’ function in the ‘stats’ package R (version 3.6.1). In consequence, a large number of columns (14190, 3023, and 15893) from each gene expression dataset were dropped, but the total number of distinct gene symbols has not changed and genetic significance also remains the same.

### Significance analysis of microarray

Significance analysis of microarray (SAM) is a test method where the test statistic measures the difference in gene-expression values between control and treatment groups divided by the standard deviation of repeated measurements. It is specifically designed to address data from large microarrays, as SAM guarantees the probability of error to not increase with the number of comparisons, in contrast with an increase observed with the number of *t* tests. Once all test statistic scores are acquired, they are ranked, and genes with scores greater than an adjustable threshold delta (Δ) are selected. Subsequently, the false discovery rate (FDR) is computed to identify the rate of genes incorrectly identified as significant by using the permutations of the measurements [16]. In this study, we performed the SAM tests in the ‘siggenes’ package R (version 3.6.1). Total permutations (1000) and FDR = 0.0005 were used for both cohorts.

### Additional *t* test and correlation analysis

Using the GSE98278 dataset which contains stability (stable vs. ruptured) and size (intermediate vs. large) information of AAA, we next aimed to find out the DEGs that are actively engaged in the development of AAA. Unpaired *t* tests were implemented to 12 commonly significant genes from two previous SAM tests, and the resulting *P*-values were corrected with Benjamini–Hochberg procedure (FDR = 0.25). Additionally, we conducted correlation tests between two genes of our main focus, Frizzled-related protein (*FRZB*) and chromosome 1 open reading frame 24 (*C1orf24*), and the other genes in each cohort (GSE7084, GSE57691) for gene set analysis. |R| > 0.7 and *P*<0.05 were used as cut-off values. To obtain the signaling pathways, correlated genes with *FRZB* and *C1orf24* were subjected to Reactome pathway analysis using Enrichr (https://amp.pharm.mssm.edu/Enrichr/) [15].

### Maintenance of zebrafish and morpholino injection

Wild-type AB zebrafish and *fli1*; DsRed transgenic zebrafish were maintained in an automatic circulation system (Genomic-Design, Daejeon, Korea) at 28.5°C [16]. Every experiment using zebrafish embryos were performed in accordance with the guidelines of the Ulsan National Institute of Science and Technology (UNIST) Institutional Animal Care and Use Committee (IACUC) (IACUC approval number: UNISTIACUC-15-14, date: 2016-10-11) at UNIST building 103. Zebrafish embryos for experiments were cultured using E3 solution in incubators at 28°C. A translation-blocking morpholino (MO) targeting *frzb* (Gene Tools, Philomath, U.S.A.) was resolved in DEPC water in 25 ng/nl stock. The sequence of frzb ATG MO is 5′-AGCGGAGTTGATAGAAGAATGACAT-3′. The morpholino targeting *frzb* was injected in embryos of wild-type AB zebrafish at the one-celled stage of development. Microinjections were performed using Femtojet 4i microinjector (Eppendorf, Hamburg, Germany).

### Confocal imaging for the live zebrafish embryo

The zebrafish embryos were anesthetized using 0.02% tricaine and mounted with 3% methyl cellulose for imaging. Then, embryos were observed with a confocal microscope (LSM880, Carl Zeiss, Oberkochen, Germany).

## Results

### Data characteristics

To evaluate candidate genes of progression of AAA, we analyzed three cohorts, specifically GSE57691, GSE7084, and GSE98278. For GSE57691, gene expression was assessed in aortic biopsies from ten patients that were organ donors non-AAAs and 49 patients diagnosed with AAAs (mean AAA diameter: 62.3 ± 10.2 mm) (Table 1). AAA patients were less likely to be female compared with the non-AAA patients (small AAA: 0%, large AAA: 7%, non AAA: 40%; *P*<0.05). For GSE7084, aortic wall samples were collected from 10 AAA patients who underwent repair operation for AAA and from 11 non-AAA autopsies. The non-AAA samples from GSE57691 and GSE7084 were defined an abdominal aorta with size of under 50 mm. Of the ten patients experiencing AAA, four samples were used for further analysis along with another four aortic samples acquired from the non-AAA cohort. These were all clustered as a pool in the raw data using average statistics. Specifically, we disassembled the two pools to allow for a correct representation of the sample size. For GSE98278, the cohort consisted of 48 patients undergoing elective (stable AAA, *n*=31) and emergency (ruptured AAA, *n*=17) treatment. The stable AAA and the ruptured AAA groups exhibited differences with regard to age (69.5 ± 7.2 vs 73.5 ± 11.3; *P*<0.05), sex (3 vs 23% female sex; *P*<0.05), and AAA diameter (62.3 ± 12.1 vs 77.0 ± 14.7; *P*<0.05). Within the stable AAA group, the patients were divided according to the size of their AAAs into intermediate (54.1 ± 1.8 mm) and large (84.1 ± 12.6 mm) subgroups.

### Identification of genes associated with AAA progression

**Figure 1:**
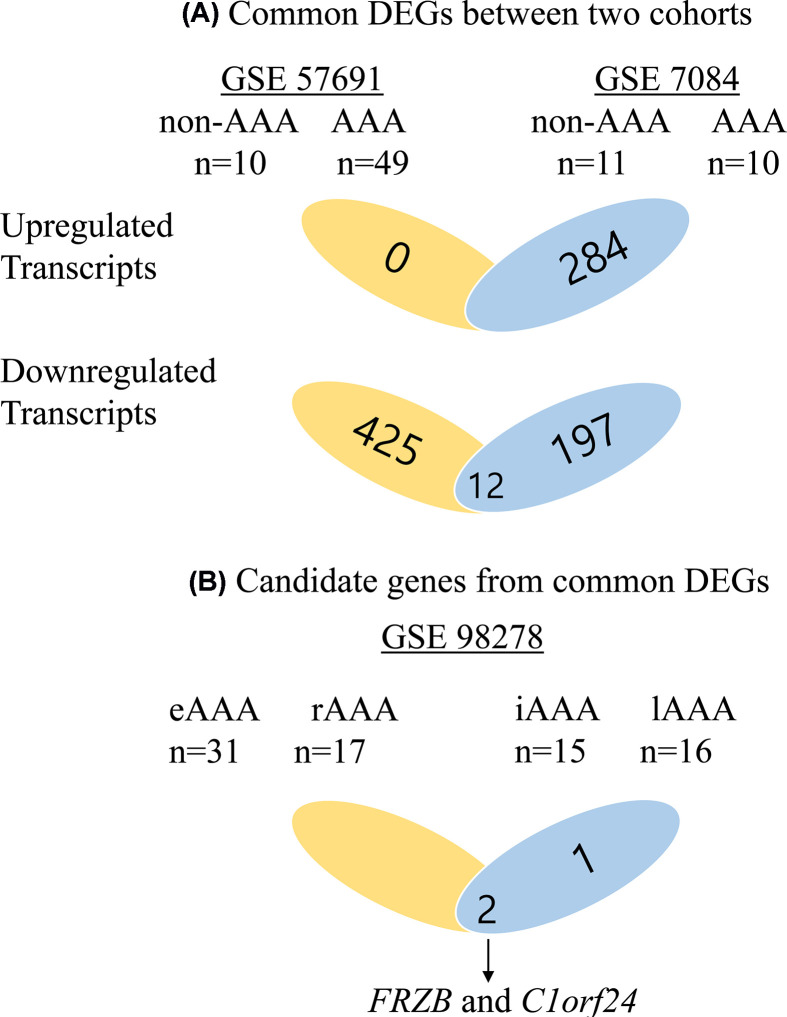
Flow charts of study design (**A**) Up-regulated transcripts were 0 from GSE57691 and 284 from GSE7084. Down-regulated transcripts were 438 from 57691 and 208 from GSE7084. Twelve genes were significant in both cohorts. (**B**) Of these 12 genes, 3 were significant between intermediate size of AAA and large size of AAA in GSE 98278; 2 were significant between stable AAA and rupture AAA in GSE 98278. *FRZB* and *C1orf24* were concomitantly significant. Abbreviations: eAAA, elective AAA; iAAA, intermediate AAA; lAAA, large AAA; rAAA, ruptured AAA.

**Figure 2:**
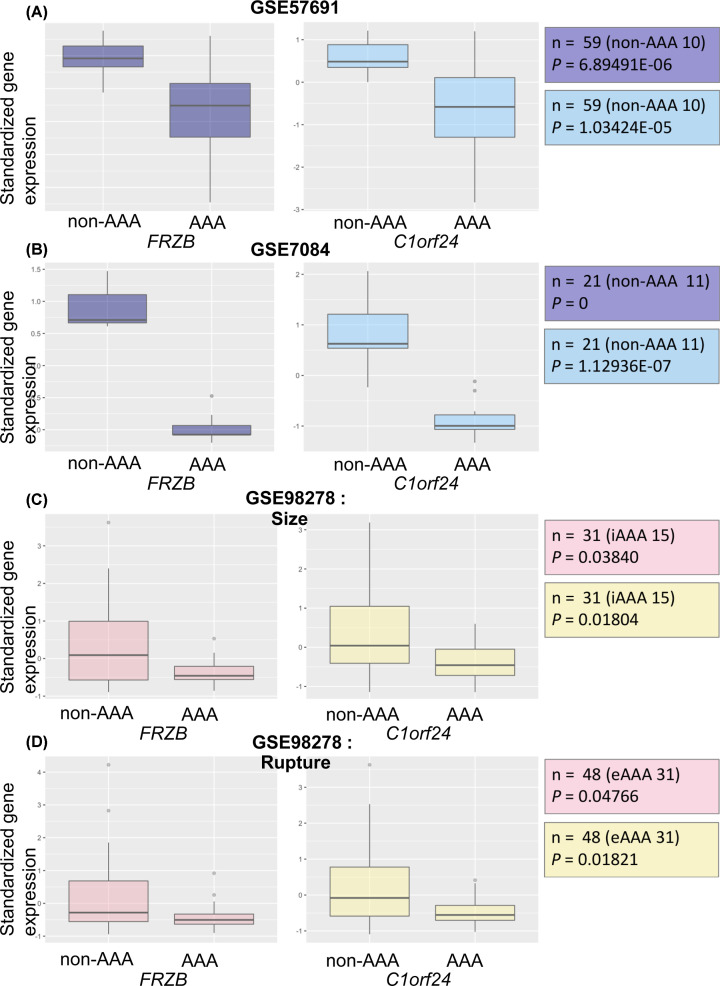
Box plots for the expression values of *FRZB* and *C1orf24* (**A**) The expression values of between *FRZB* and *C1orf24* between AAA patients (*n*=49) and non-AAA patients (*n*=10) from GSE 57691. *FRZB* and *C1orf24* were significantly down-expressed in AAA patients. (**B**) The expression values of *FRZB* and *C1orf24* between AAA patients (*n*=10) and non-AAA patients (*n*=11) from GSE 7084. *FRZB* and *C1orf24* were significantly down-expressed in AAA patients. (**C**) The expression values of *FRZB* and *C1orf24* between intermediate size AAA (*n*=15) and large size AAA (*n*=16) from GSE 98278. *FRZB* and *C1orf24* were significantly down-expressed in large size AAA. (**D**) The expression values of *FRZB* and *C1orf24* between eAAA (*n*=31) and rAAA (*n*=17) from GSE 98278. *FRZB* and *C1orf24* were significantly down-expressed in ruptured AAA. Abbreviations: eAAA, elective AAA; iAAA, intermediate AAA; lAAA, large AAA; rAAA, ruptured AAA.

We performed SAM to identify DEGs within each cohort, and the study cohorts included GSE57691, GSE7084 divided into two groups, non-AAAs and AAAs. A total of 436 down-regulated genes were identified from GSE57691, and 284 up-regulated and 209 down-regulated genes were identified from GSE7084 (Figure 1). A total of 12 DEGs were statistically significant in both cohorts. DEGs are listed in Supplementary Table S1. Of these 12 genes, *t* test analysis was performed on GSE98278 to show the difference of expression in two subgroups, intermediate size and large size and stable AAA and ruptured AAA. Three genes (*C1orf24, FRZB*, and *KIAA0367*) and two genes (*C1orf24* and *FRZB*) were differentially expressed at the first subgroup and second subgroup, respectively. *C1orf24* and *FRZB* were shown to be simultaneously altered in a statistically significant manner as the size and instability of AAA increased (Figure 1). The mRNA expression of the *C1orf24* and *FRZB* genes was decreased in AAA patients from GSE57691 and 7081 (Figure 2A,B) and with increasing size and instability of AAA from GSE982789 (Figure 2C,D).

### Correlation analysis

To identify the pathway correlated with *FRZB* and *C1orf24*, we performed Pearson’s correlation of *FRZB* and *C1orf24* with other genes GSE57691 and GSE7084. By applying |R| > 0.7 and *P*<0.05, 64 genes were positively correlated with *FRZB* simultaneously from GSE57691 and GSE7084. Seventy-one genes were positively correlated with *C1orf24*, and three genes were negatively correlated with *C1orf24* from two cohorts (Supplementary Table S2). Correlated genes with *FRZB* and *C1orf24* were subjected to Reactome pathway analysis using Enrichr. With *FRZB*, the pathways were associated with RHO GTPase and elastin fiber formation (Table 2). With *C1orf24*, the pathways were elastic fiber formation, extracellular matrix organization, and cell–cell communication (Table 3).

**Table 2:** Top ten significantly enriched Reactome pathways with *FRZB* and correlated genes from GSE57691 and GSE7084 using Enrichr (https://amp.pharm.mssm.edu/Enrichr/)

Term	Overlap	*P*-value	Odds ratio	Combined score	Genes
**RHO GTPases activate ROCKs**	3/17	2.16E-05	54.29864253	583.4084401	*PPP1CB; MYH11; MYH10*
**RHO GTPases activate PAKs**	3/21	4.18E-05	43.95604396	443.2037946	*PPP1CB; MYH11; MYH10*
**Molecules associated with elastic fibers**	3/30	1.25E-04	30.76923077	276.5468079	*MFAP4; ITGA8; FBLN5*
**Elastic fiber formation**	3/41	3.20E-04	22.51407129	181.1923658	*MFAP4; ITGA8; FBLN5*
**RHO GTPases activate PKNs**	3/60	9.82E-04	15.38461538	106.5481366	*PPP1CB; MYH11; MYH10*
**RHO GTPases activate CIT**	2/16	0.001211888	38.46153846	258.2913719	*MYH11; MYH10*
**Semaphorin interactions**	3/67	0.001353045	13.77726751	91.00432898	*ITGA1; MYH11; MYH10*
**Integrin cell surface interactions**	3/67	0.001353045	13.77726751	91.00432898	*ITGA1; ITGA8; JAM3*
**Extracellular matrix organization**	5/283	0.002262817	5.436259853	33.11304623	*MFAP4; ITGA1; ITGA8;FBLN5; JAM3*
**Sema4D induced cell migration and growth-cone collapse**	2/24	0.00274105	25.64102564	151.2670332	*MYH11; MYH10*

**Table 3:** Top ten significantly enriched Reactome pathways with *C1orf24* and correlated genes from GSE57691 and GSE7084 using Enrichr (https://amp.pharm.mssm.edu/Enrichr/)

Term	Overlap	*P*-value	Odds ratio	Combined score	Genes
**Elastic fiber formation**	3/41	4.32E-04	20.32520325	157.4485645	*MFAP4; EFEMP1; LOXL4*
**Extracellular matrix organization**	6/283	5.44E-04	5.889281508	44.26820723	*MFAP4; EFEMP1; ITGA1; LOXL4; COL8A1; JAM3*
**Cell–cell communication**	4/131	0.001281745	8.481764207	56.48458548	*CLDN12; CTNNA1; CDH13; WASL*
**Cell–cell junction organization**	3/61	0.001386	13.6612	89.90722	*CLDN12; CTNNA1; CDH13*
**RHO GTPases activate ROCKs**	2/17	0.001678	32.67974	208.8211	*PPP1R12A; MYH10*
**Semaphorin interactions**	3/67	0.001817	12.43781	78.49063	*DPYSL3; ITGA1; MYH10*
**RHO GTPases activate PAKs**	2/21	0.002568	26.45503	157.7983	*PPP1R12A; MYH10*
**Cell junction organization**	3/86	0.003697	9.689922	54.26647	*CLDN12; CTNNA1; CDH13*
**Molecules associated with elastic fibers**	2/30	0.005209	18.51852	97.35994	*MFAP4; EFEMP1*
**Adherens junctions interactions**	2/31	0.005555	17.92115	93.06567	*CTNNA1; CDH13*

### *frzb* is essential for the development of zebrafish embryo through regulation of vascular integrity

**Figure 3:**
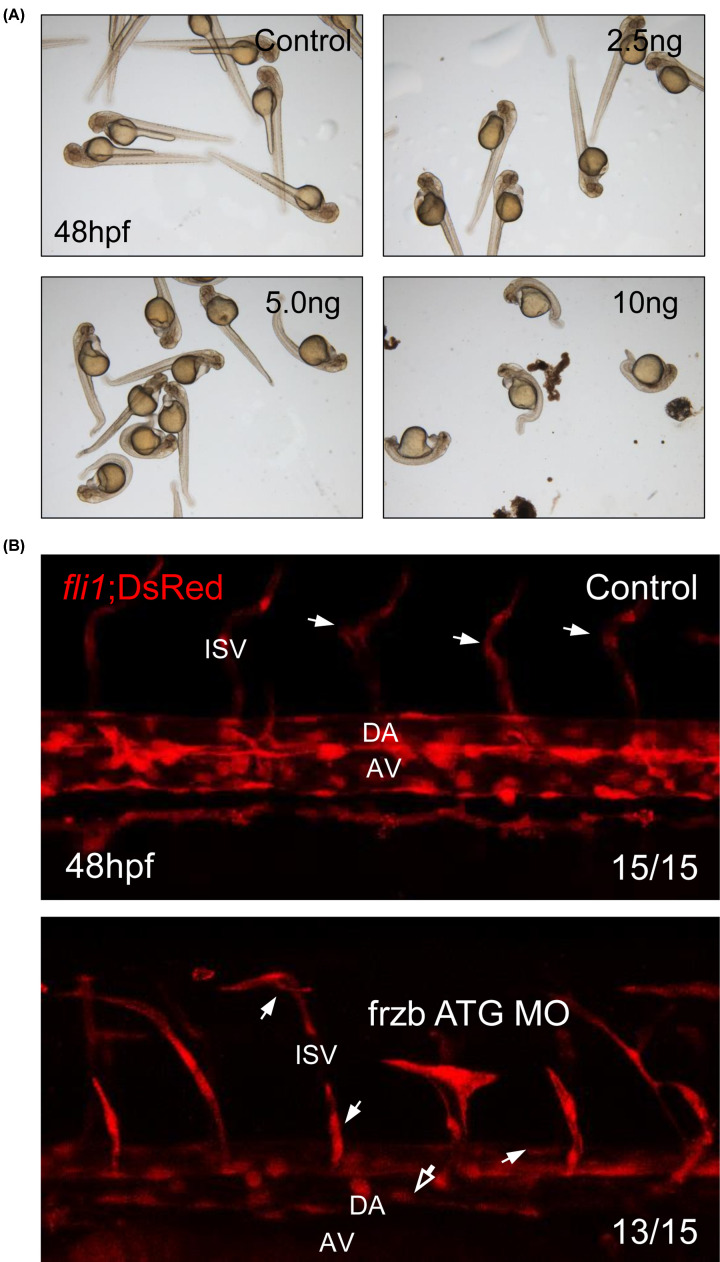
*frzb* is essential for the development of zebrafish embryo through regulation of vascular integrity (**A**) Imaging of live embryos after morpholino injection (0, 2.5, 5, and 10 ng). (**B**) Confocal imaging of *fli1*; DsRed transgenic zebrafish embryos from control embryos and frzb ATG MO-injected embryos. Abbreviations: AV, axial vein; DA, dorsal aorta; hollow arrow, narrow dorsal aorta and axial vein; ISV, intersomite vessel. White arrow, abnormal branch of intersomite vessel.

RHO GTPase pathway and elastic fiber formation-related genes are analyzed from correlation analysis, and those genes are associated with vascular integrity. *C1orf24* was not evolutionally conserved in the vertebrates, whereas *FRZB* was conserved. Alignment between human *FRZB* and zebrafish *frzb* shows 70% exactly same residue, 12% similar structure of residue, and 6% gaps in compositional matrix adjustment (Supplementary Figure S1). Considering these data, we validated our correlation data from *FRZB* by observing vascular formation of zebrafish embryos. To investigate the function of frzb in vascular integrity, knockdown of *frzb* was processed using MO injection. Dependent on dosage of frzb ATG MO, development of zebrafish embryos was interrupted (Figure 3A). Since development of embryos with 5 and 10 ng were highly interrupted and developmental defects were shown from 2.5 ng of MO-njected group, we decided 2.5 ng is enough for knockdown.

*Frzb* is highly correlated with elastic fiber formation-related genes at patient database (Table 2), and elastic fiber is important for the vascular integrity. To validate our informatics data, vascular formation of zebrafish embryos was checked using *fli1*;DsRed transgenic zebrafish. As many zebrafish embryos with defects in vascular integrity showed abnormal vascular formation [17,18], frzb ATG MO-injected embryos show disrupted vascular structure in trunk. Lumen of dorsal aorta and axial vein became narrow, and abnormal branch of intersomite vessels were observed (Figure 3B). These data suggest that *frzb* is essential for the vascular integrity.

## Discussion

Even though multiple etiological, genetic factors contributing to AAA, as well as its pathobiology, have been extensively studied, the mechanisms of progression of AAA are still incompletely understood. Through analysis of three gene expression studies, we tried to narrow down possible candidate genes that provide a comprehensive view of the disease.

Through literature review, we had found three articles which analyzed DEGs of AAA from datasets in GEO [19–21]. The datasets used from each articles were as follows; Shen et al. [19], GSE47472; Wan et al. [20], GSE7084, GSE47472, and GSE57691; Chen et al. [21], GSE7084, GSE47472, and GSE57691. The tissue samples in GSE47472 were non-aneurysmal aortic neck tissues, so we considered the data in GSE47472 were not suitable for our study design. Because the data in GSE98278 included important clinical data such as size of AAA and rupture status of AAA, we analyzed the data of GSE98278 with other two datasets, GSE7084 and GSE57691.

By applying very low FDR value (0.0005) in analysis of DEGs from two cohorts (GSE7084 and GSE57691), only 12 genes were identified. The major limitation of microarray-based mRNA expression profiling is that tissue samples from end-stage disease provide little information about initiation [22]. The only option for vascular surgeons to retrieve tissue samples of AAA is through open surgeries of AAA, in which diameter is mostly over 50 mm. In our study, the mean diameter of AAA from GSE57691 is 62.3 mm. By comparing disease-progressed tissue samples and normal tissues, it is non-logical to develop DEGs involving initiation of AAA. Therefore, 12 DEGs from GSE 7084 and GSE 57691 were analyzed to identify which genes were involved in terminal AAA and rupture. Only two genes, *FRZB* and *C1orf24*, were differentially expressed along size expansion and rupture. *FRZB* binds to both Wnt-8 and Wnt-1 and acts as a functional inhibitor of Wnt-8 activity in *Xenopus* embryos [23]. *FRZB* has been reported as a key factor of skeletal morphogenesis and osteoarthritis [24,25]. *C1orf24* contains a DnaJ motif, a feature of heat shock protein that was investigated as a factor associated with renal carcinogenesis [26]. It was also reported that *C1orf24* was up-regulated in thyroid cancer and might increase proliferation and cell migration [27].

To evaluate which of the molecular pathways were related to *FRZB* and *C1orf24*, we performed correlation analysis on GSE 7084 and GSE57691. Then, pathway analysis with correlated genes revealed correlated genes with *FRZB* and *C1orf24* genes are involved in the pathway of RHO GTPase and elastin fiber formation. In many experimental studies, RHO GTPase played central roles in vascular smooth muscle cell proliferation, migration, and contractility, differentiation, and ROS generation [28–32]. In rats with balloon‐injured arteries, increased RhoA/ROCK activity contributes to neointimal formation, and these detrimental effects are significantly suppressed by the ROCK‐specific antagonist Y27632 [33]. Regarding VSMC differentiation, through modulating serum response factor-dependent skeletal and smooth muscle gene expression, the RhoA/ROCK signaling pathway regulates VSMC differentiation [34]. The important characteristics of aneurysmal wall are breakdown of elastin and collagen, VSMC apoptosis, and activation of immune responses [35]. We showed *FRZB* and *C1orf24* correlated with RHO GTPase and elastin fiber formation pathways. Among them, we validated that *FRZB* is essential for the vascular integrity using zebrafish embryos. That means low expression of *FRZB* is associated with the progression of AAA by diminishing integrity of abdominal aorta.

Many studies have validated the candidate genes of AAA pathobiology through hypothesis-driven way. These studies have been on a ‘best guess’ basis and do not fit with the studies of diseases for which underlying pathological processes are unclear [36]. With advances in genetic technology and bioinformatics, unbiased high throughput genome-wide association studies (GWASs) have widely made. In meta-analysis of GWASs for AAA, four new AAA risk loci, rs1795061 (*SMYD2*), rs9316871 (*LINC00540*), rs3827066 (*PCIF1/MMP9/ZNF335*), and rs2836411 (*ERG*), were identified, as well as five of the six previous AAA genetic associations, rs602633 (*PSRC1-CELSR2-SORT1*), rs4129267 (*IL6R*), rs10757274 (*CDKN2BAS1/ANRIL*), rs10985349 (*DAB2IP*), and rs6511720 (*LDLR*) [37]. One study used VSMCs isolated from patients with AAA and controls to assess DNA methylation in the genes found within the nine associated loci, and altered DNA methylation levels were found in three genes (*ERG, IL6R*, and *SMYD2*) [38]. Despite their highly significant associations with AAA, these genetic loci explain only a small proportion of the heritability of AAA.

Even though microarray-based mRNA expression profiling has limitations described before and difficulties, it can a useful tool in dissecting disease pathogenesis, especially when pathogenesis of disease is not well established. Among three cohorts we analyzed, Lenk et al. (GSE 7084) compared whole genome expression profile between AAA (*n*=10) and normal (*n*=10) and showed broad coordinate gene expression in immunological pathways [39]. *FRZB* was one of the top 100 most differential genes in Supplementary Table S2. Biros et al. (GSE 57691) used AAA tissue samples for analyzing expression difference between AAA and aortic occlusive disease [40]. Direct comparison of our study with GSE57691 is impossible; however, *FRZB* and C*1orf24* was down-regulated in AAA group. Gäbel et al. (GSE98278) tried to validate the candidate genes relating to terminal AAA [41]. Based on the studies that molecular mechanisms and genetic factors might differ in initiation, growth, and rupture of AAA [42], they developed ten DEGs and showed that these genes converged at activation of HIF-1α network. They also showed histologic quantification of angiogenesis in ruptured AAA. Even though it might provide important insight of terminal AAA, by not comparing normal aortic tissue, genes involving process of AAA development might be neglected. Therefore, we tried to narrow down the genes comparing AAA tissues and normal aortic tissue, then analyzed on two groups, size difference and rupture. We considered this analytic strategy is more logical to develop candidate genes of AAA progression.

To prove the protein level of candidate genes in AAA patients, the optimal tissue for our purpose was aneurysmal tissue retrieved from open surgery of AAA. In the Biobank of our country, however, there was no tissue sample of AAA. This is our major limitation. Further investigations will be held to validate the correlation of these pathways with our candidate genes.

## Conclusions

The present study examined the candidate genes involved in the progression of AAA. We were able to identify two gene expressions (*FRZB* and *C1orf24*) by analyzing three cohorts. In pathway analysis with correlated genes with these two candidate genes (*FRZB* and *C1orf24*), we showed the pathways were related to VSMC and elastin fiber formation, which might imply these candidate genes are involved in aneurysm integrity. Additionally, we validated that *FRZB* was linked to the vascular integrity using zebrafish embryos. Considering our findings, we believe that *FRZB* is a possible candidate gene involved in AAA progression.

## Supplementary Material

Supplementary Figure S1

Supplementary Tables S1-S2

## Data Availability

The data that support the findings of the present study are available from the corresponding author upon reasonable request.

## Abbreviations

AAA: abdominal aortic aneurysm
*C1orf24*: chromosome 1 open reading frame 24
DEG: differentially expressed gene
DEPC: diethylpyrocarbonate
FDR: false discovery rate
FRZB: Frizzled-related protein
GEO: Gene Expression Omnibus
GWAS: genome-wide association study
HIF-1α: hypoxia-inducible factor 1 alpha
IACUC: Institutional Animal Care and Use Committee
MO: morpholino
NCBI: National Center for Biotechnology Information
ROCK: Rho-associated protein kinase
SAM: significance analysis of microarray
UNIST: Ulsan National Institute of Science and Technology
VSMC: vascular smooth muscle cell
