# Supplementary material for: FRZB as a key molecule in abdominal aortic aneurysm progression affecting vascular integrity
Source: Biosci Rep. 2021 Jan 6;41(1):BSR20203204. doi: 10.1042/BSR20203204 (PMC7789806; doi:10.1042/BSR20203204)
Supplement: Supplementary Figure S1 [file BSR-2020-3204_supp.pdf]

Score	Expect	Method	Identities	Positives	Gaps
446 bits(1148)	1e-163	Compositional matrix adjust.	210/301(70%)	248/301(82%)	19/301(6%)

```
Query  8    GMLLLRAGLLALAALCLLRVP-GARAAACEPVRIPLCKSLPWNMTKMPNHLHHSTQANAI  66
             M      +  LA  CLL +P G  AA+CEP+RIP+CKS+PWNMTKMPNHLHHSTQANA+
Sbjct  3    NMFSYEMFICVLAFACLLEIPRGTGAASCEPIRIPMCKSMPWNMTKMPNHLHHSTQANAV  62

Query  67   LAIEQFEGLLGTHCSPDLLFFLCAMYAPICTIDFQHEPIKPCKSVCERARQGCEPILIKY  126
            LAIEQFEGLLGT CS DLLFFLCAMYAPICTIDFQH+PIKPCKSVCERA+ GCEP++ +Y
Sbjct  63   LAIEQFEGLLGTQCSADLLFFLCAMYAPICTIDFQHDPIKPCKSVCERAKCGCEPVMKRY  122

Query  127  RHSWPENLACEELPVYDRGVCISPEAIVTADG-----------------ADFPMDSSNGN  169
             H+WPE+LACEELPVYDRGVCISPEAIV A+G                  DFPMDS N N
Sbjct  123  NHTWPESLACEELPVYDRGVCISPEAIVKAEGPDNSYYQDPAKCNPEGNPDFPMDSHNTN  182

Query  170  CRGASSERCKCKPIRATQKTYFRNNYNYVIRAKVKEIKTKCHDVTAVVEVKEILKSSLVN  229
            C+GA ++RCKCK ++  QKTY +NNYNYVIRA+VKEI+ + HD++A+VEVKE+LKSSLVN
Sbjct  183  CKGA-NDRCKCKSVKFNQKTYSKNNYNYVIRARVKEIRIRNHDLSAIVEVKEVLKSSLVN  241

Query  230  IPRDTVNLYTSSGCLCPPLNVNEEYIIMGYEDEERSRLLLVEGSIAEKWKDRLGKKVKRW  289
            IPRDTV LY +SGCLCPPL  N+EYIIMGYE+EERSRLLL++ SIA+KWK+++G+KVKRW
Sbjct  242  IPRDTVTLYYNSGCLCPPLTANDEYIIMGYENEERSRLLLIDRSIAQKWKEKIGRKVKRW  301

Query  290  D  290
            D
Sbjct  302  D  302
```

Supplementary Figure 1. *frzb* is evolutionally conserved in the vertebrate. Alignment of amino acid sequence between human *FRZB* (Query) and zebrafish *frzb* (Subject).
